# A Comprehensive Management of Neck Hematoma in Post-Thyroidectomy Patient for Papillary Thyroid Cancer: A Case Report

**DOI:** 10.7759/cureus.42689

**Published:** 2023-07-30

**Authors:** Keval Thakkar, Nkiruka Lauretta Nwangene, Reeju Maharjan, Sandra Francis, Carlo Kristian C Carredo, Rojaj Dahal, Aadil Khan

**Affiliations:** 1 Department of Internal and Hospital Medicine, Moffitt Cancer Center, Tampa, USA; 2 Department of Internal Medicine, Larkin Community Hospital, South Miami, USA; 3 Department of Internal Medicine, Caribbean Medical University, Willemstad, CUW; 4 Department of Neurology, V.N. Karazin Kharkiv National University, Kharkiv, UKR; 5 Department of Medicine, Windsor University School of Medicine, Cayon, KNA; 6 Department of General Surgery, Cebu Institute of Medicine, Cebu, PHL; 7 Department of Internal Medicine, Manipal College of Medical Sciences, Pokhara, NPL; 8 Department of Cardiology, University of Illinois Chicago, Chicago, USA

**Keywords:** papillary carcinoma, obstruction, thyroid, hematoma, thyroidectomy

## Abstract

A post-thyroidectomy hematoma is a rare, potentially fatal surgical complication that may present as hoarseness, dysphagia, and difficulty in breathing, which could progress to complete airway obstruction and, ultimately, death. The treatment for a neck hematoma is emergent surgical drainage. While certain precautions can be taken to prevent this complication, such as the cessation of any anticoagulants prior to surgery, it is still a feared complication of thyroidectomy with an increasing prevalence. In this paper, we discuss a case of a 62-year-old female with papillary thyroid cancer who presents with a postoperative complication of a neck hematoma requiring emergent surgery and conduct a literature review on managing post-thyroidectomy hematomas.

## Introduction

The increasing prevalence of thyroid disease and thyroid surgeries have raised concerns regarding potential complications associated with thyroidectomy. While the procedure itself is generally considered safe, there is a risk of postoperative complications that can be highly alarming [1]. While recurrent laryngeal nerve injury and permanent hypothyroidism, hyperthyroidism, hypoparathyroidism, hypocalcemia, and surgical site infections are well-recognized complications, one frequently overlooked yet potentially fatal complication is postoperative neck hematoma (PNH) [2]. PNH refers to the accumulation of blood in the neck area following thyroidectomy or any surgery involving the neck. It is one of the potential complications of thyroidectomy and can have various consequences if not promptly diagnosed and treated. The severity of the consequences depends on the size of the hematoma and its effect on surrounding structures. PNH can lead to airway compression, resulting in acute respiratory distress or even death, requiring immediate surgical intervention [3]. The prominence of neck hematoma as the primary cause for reoperation emphasizes the critical importance of close monitoring and early intervention during post-thyroidectomy [4]. The occurrence of postoperative hematoma is estimated to range from approximately 0.1% to 1.1% depending on surgical technique, patient characteristics, or comorbidities [5].

Additionally, it is crucial to recognize the risk of acute airway distress when patients are in a supine position, necessitating preparedness for emergency intubation. The treatment of hematoma largely depends on the symptoms exhibited, with more cases requiring exploration and evacuation while immediate bedside evacuation is only necessary for unstable or progressively worsening hematomas in patients [6]. Other treatment approaches include manual aspiration. Use of hemostatic agents, blood transfusion in case of massive bleeding, and use of compression stockings. Achieving hemostasis remains the primary objective after preserving vital structures during thyroid surgery. To minimize morbidity and enhance the safety of thyroidectomy, it is imperative to develop a comprehensive understanding of its complications. This case report aims to investigate the occurrence, management, and impact of postoperative hematoma, shedding light on strategies to improve patient outcomes and ensure the surgical triumph of thyroidectomy.

## Case presentation

A 62-year-old woman with a history of hypothyroidism presented with an enlarged right solitary thyroid nodule measuring greater than 5ccm at the General Surgery (GS) Division of Georgetown University Hospital, a high-volume center for thyroidectomies. She was diagnosed with hypothyroidism and was compliant with levothyroxine. The patient reported that over the last eighteen months, she felt her thyroid gland enlarge. She denied having had prior ultrasound-guided fine needle aspiration (FNA) before. There was no history of swallowing or breathing difficulty, hoarseness, or radiation to the head and neck region in the past and no history of hypertension or anticoagulation. She denied a family history of other endocrinopathies. Her recent thyroid profile was within normal range, and she was on levothyroxine 1.7 mcg/kg daily. 

On examination, she was hemodynamically stable and afebrile. Local examination revealed a palpable solitary right thyroid nodule measuring 2.5 x 2.2 cm with overlying warm, dry, and non-tender skin. The rest of the systemic examination was unremarkable.

**Figure 1:**
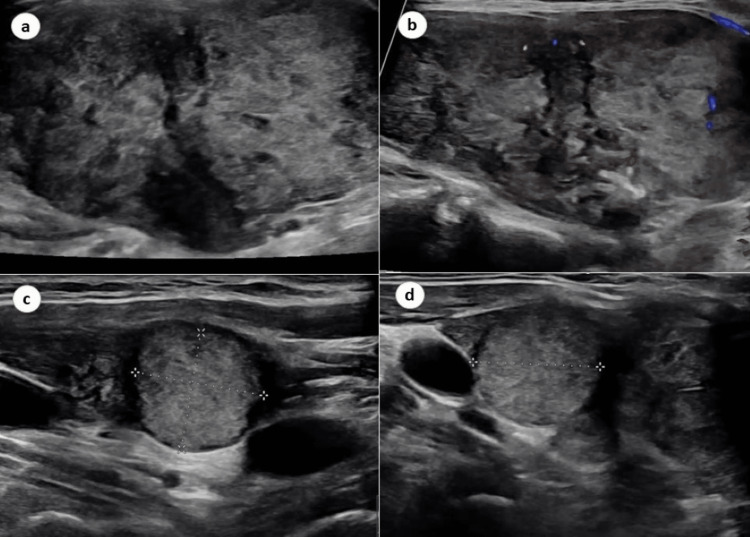
Ultrasound of the thyroid gland demonstrating (a, b) right lobe (5.7 x 2.8 x 4.4 cm) and the left lobe (1.4 x 0.3 x 0.6 cm), (c) a right lateral lower pole isoechoic solid and cystic nodule measuring 1.6 x 1.4 x 1.5, and (d) a right mid pole mixed echogenicity solid and cystic nodule measuring 5.3 x 2.7 x 4.3 cm.

Her blood and biochemical investigations were within normal range except for thyroid function tests which include thyroid stimulating hormone (TSH) of 0.1 mlIU/L (0.4-4). Ultrasound of the thyroid gland was performed which revealed that the right lobe measured 5.7 x 2.8 x 4.4 cm and the left lobe measured 1.4 x 0.3 x 0.6 cm. The isthmus measured 0.1 cm. Additionally, a right lateral lower pole isoechoic solid and cystic nodule measuring 1.6 x 1.4 x 1.5 cm and a right mid pole mixed echogenicity solid and cystic nodule measuring 5.3 x 2.7 x 4.3 cm were noted (Figure 1). No immediate extrathyroidal abnormality was found. Owing to the above finding, FNA of the mid pole nodule was performed, and the biopsy revealed that the patient had papillary thyroid carcinoma, Bethesda VI, and subsequently, lymph node mapping with FNA was conducted, which showed that there were suspicious right level six (VIa) and paratracheal lymph nodes. However, the final pathology revealed no evidence of metastatic disease.

Following the above diagnosis and consult with the GS team, total thyroidectomy and right central compartment neck dissection were planned. The patient was taken to the operating room and placed in a supine position. The patient underwent a thyroidectomy with central compartment neck dissection for cervical lymphadenopathy. The procedure was performed under general anesthesia with the patient supine. A transverse cervical incision was made, and subplatysmal flaps were raised. The right thyroid lobe was extensively adherent to the strap muscles but was successfully medialized, and the superior and inferior pole vessels were divided. Middle thyroid vein was properly ligated and external branch of superior laryngeal nerve and parathyroids safeguarded and preserved with intact vascularity. The central compartment neck was dissected, and the recurrent laryngeal nerve was protected. The left thyroid lobe was smaller and removed without a central compartment neck dissection. The strap muscles were reapproximated with Vicryl sutures, and the wound was closed with Monocryl. The suction drain was inserted, and the patient was extubated and transferred to the recovery room in stable condition.

She had just been brought to the recovery room when swelling identified in the neck region led to a clinical concern for neck hematoma. She was extubated without nausea or vomiting and had no retching or coughing. She started experiencing swelling six hours after surgery with severe pain and neck stiffness and mild dyspnea. Thus, the decision was made to return to the operating room emergently to investigate and address the issue. The surgical team reopened her previous incision to decompress the swelling and facilitate airway access. Once they intubated her, she was transferred to the operating room table, and her arms were tucked away, pads were secured, and a temperature probe was inserted. The surgical team then sterilely prepped and draped the area and performed a surgical timeout. The previous incision was fully opened and inspected, and blood clots were evacuated from the superficial neck space. There was no active bleeding site, so the surgeons turned their attention to opening the deeper neck space. They paid attention to the left and right sides of the neck, carefully inspecting and irrigating the area to ensure there was no source of active bleeding. On the right side, they clamped and tied off a small vein, a tributary of internal jugular vein located posterior laterally, which was the only potential culprit of the cause of the patient's bleeding.

The surgical team inspected the neck thoroughly and asked the anesthesiologists to perform a Valsalva maneuver a few times. After ensuring hemostasis, a small amount of Surgicel was placed in the wound, and a 15 French round Blake drain was inserted, secured in place, and connected to close bulb drainage. The experts reapproximated the strap muscles and the platysma with buried, interrupted 3-0 Vicryl sutures. Deep dermal sutures were also placed with buried, interrupted 3-0 Vicryl. Finally, the surgical team closed the skin with a running knotless 4-0 Monocryl and dressed the wound with sterile strips. Before concluding the surgery, all surgical counts were verified, and the wound was cleaned and dried. The anesthesia team extubated the patient without incident, and she was transferred to the recovery room in stable condition. The operating surgeon was present and scrubbed for the entire operation. Overall, the surgical team thoroughly examined the patient's neck and ensured that all potential sources of bleeding were addressed. During the patient's examination, there were no signs of bleeding present. The intraoperative/hospital course and findings were reviewed with the patient, and the necessary postoperative expectations and instructions were provided to them. The patient was advised on caring for their wound and recommended gentle range of motion exercises to the neck and ambulation as tolerated.

Concerning signs and symptoms were discussed with the patient, and they were informed about when they should seek medical attention. Hypocalcemic symptoms were also reviewed, and the associated plan was discussed. Additionally, the patient was instructed to continue supplemental multivitamin and to have the drain removed. Follow-up plans were reviewed with the patient, and any questions or concerns they had been addressed. Overall, the patient has received a comprehensive review of their status and has been given appropriate guidance for their postoperative recovery.

## Discussion

The thyroid gland is a highly vascularized organ; thyroidectomy is a common and relatively safe procedure. However, like other procedures, there is a risk of postoperative complications. Hypoparathyroidism, recurrent laryngeal nerve paralysis, loss of high-pitched voice, hematoma, and seroma are well-known post-thyroidectomy complications [7]. Among these, hypocalcemia is considered the most common cause of hospital readmission, and hematoma is the most frequent indication for reoperation. Although uncommon, a post-thyroidectomy hematoma is a serious, potentially life-threatening complication with an incidence of 0.7% to 4.7% and can reportedly reach 6.5% [8]. This is extremely important when considering ambulatory thyroidectomy. Post-thyroidectomy hematoma formation may be related to patient predisposition, surgical technique, and thyroid pathology. Post-thyroidectomy hematoma can present as a swelling and expansion of the neck, ecchymosis over the surgical site, respiratory distress, stridor, or difficulty breathing, dysphagia, hoarseness or voice changes, palpable mass in the neck, hemodynamic instability in severe cases. In severe cases, cerebral anoxia with major neurological complications and even death may occur [9].

Post-thyroidectomy hematoma commonly occurs within the first six hours after surgery; however, it can occur up to 24 hours after surgery. The risk factors for post-thyroidectomy hematoma include coexisting morbidities, including diabetes mellitus, obesity, chronic kidney disease, chronic obstructive pulmonary disease, congestive heart failure, bleeding disorder, sleep apnea, and hypertension, male sex, older age, certain thyroid diseases such as Graves' disease, malignancy, and Hashimoto's thyroiditis, retrosternal goiter, the extent of thyroidectomy, resected thyroid volume, redo-surgeries, drain use, high postoperative blood pressure, smoking, medications (antithrombotic agent), and prior neck radiation [10].

Diagnostic approaches for post-thyroidectomy neck hematoma involve a combination of physical examination, imaging modalities, and hematological investigations. Ultrasonography is often the first-line imaging modality used to evaluate post-thyroidectomy neck hematoma. It is non-invasive, readily available, and does not involve ionizing radiation. Ultrasonography can provide real-time visualization of the neck structures and identify the presence, size, and location of the hematoma. It can also help differentiate hematomas from other postoperative complications like seromas or abscesses [11]. CT scans are more sensitive and specific than ultrasound in detecting post-thyroidectomy neck hematomas. CT imaging can provide detailed cross-sectional images of the neck, allowing for a comprehensive assessment of the hematoma's size, extent, and relationship with adjacent structures. It is particularly useful when ultrasound findings are inconclusive or when there is suspicion of a deep or expanding hematoma [9,10].

**Figure 2:**
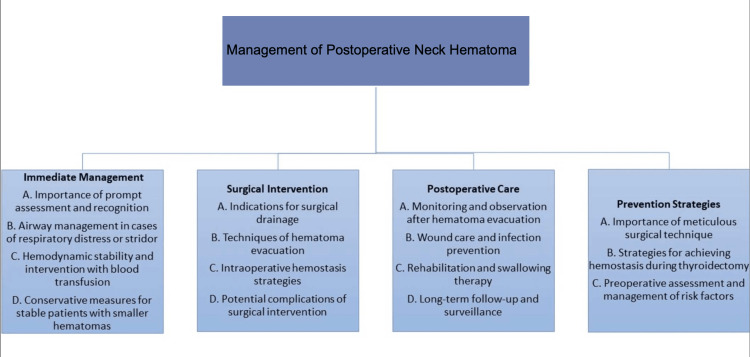
Comprehensive management of postoperative neck hematoma.

The management of PNH is explained in Figure 2 [12].

Surgical management of neck hematoma in a post-thyroidectomy patient involves emergency airway management. If the hematoma is causing airway compromise, immediate intervention is necessary. A thorough clinical assessment is performed to determine the size and extent of the hematoma. This includes physical examination, assessment of vital signs, and monitoring of the patient's overall condition. In some cases where the hematoma is small and not causing significant airway compromise, a needle aspiration may be attempted [12]. For larger hematomas or cases where aspiration is not effective, surgical evacuation becomes necessary. The patient is taken to the operating room for an emergency procedure. In the operating room, the surgeon will make an incision over the hematoma to expose it. The clot is removed, and the area is thoroughly explored to identify the source of the bleeding. Bleeding vessels are ligated or cauterized to achieve hemostasis. In some cases, hemostatic agents, such as surgical sealants or sponges, may be used to control bleeding and promote clot formation [13]. After evacuation and hemostasis, a drain may be placed in the surgical site to prevent the re-accumulation of blood and to monitor ongoing bleeding. The wound is closed in layers, and the patient is closely monitored in the recovery room and, if necessary, in the intensive care unit [14]. It is essential to identify and manage a neck hematoma as early as possible to avoid life-threatening complications. The specific approach to surgical management may vary depending on the patient's condition, the size of the hematoma, and the underlying cause of bleeding. Therefore, it is crucial that experienced surgical teams handle such cases in a timely and efficient manner [7,13].

## Conclusions

PNH is a rare, potentially fatal surgical complication that can lead to airway obstruction and death. Our case highlights the importance of early recognition and prompt intervention in managing postoperative neck hematoma after thyroidectomy. Effective collaboration among the surgical team, anesthesiologists, and critical care specialists played a crucial role in achieving a positive outcome. Close monitoring of post-thyroidectomy patients and maintaining a high index of suspicion for potential complications are vital to ensure optimal patient care.
